# Basal inferoseptal segment is highly susceptible to deformation in the clinical spectrum of transthyretin-derived amyloid cardiomyopathy

**DOI:** 10.1093/ehjopen/oeae076

**Published:** 2024-09-02

**Authors:** Toshihiro Tsuruda, Hiroshi Nakada, Yoshimasa Yamamura, Yunosuke Matsuura, Miyuki Ogata, Miyo Tanaka, Yosuke Suiko, Soichi Komaki, Hiroki Tanaka, Kohei Moribayashi, Takeshi Ideguchi, Tamasa Terada, Tomomi Ota, Keisuke Yamamoto, Kensaku Nishihira, Yoshisato Shibata, Koichi Kaikita

**Affiliations:** Cardiorenal Research Laboratory, Department of Hemo-Vascular Advanced Medicine, University of Miyazaki, 5200 Kihara, Kiyotake, Miyazaki 889-1692, Japan; Division of Cardiovascular Medicine and Nephrology, Department of Internal Medicine, University of Miyazaki, 5200 Kihara, Kiyotake, Miyazaki 889-1692, Japan; Department of Radiology, University of Miyazaki, 5200 Kihara, Kiyotake, Miyazaki 889-1692, Japan; Division of Cardiovascular Medicine and Nephrology, Department of Internal Medicine, University of Miyazaki, 5200 Kihara, Kiyotake, Miyazaki 889-1692, Japan; Division of Cardiovascular Medicine and Nephrology, Department of Internal Medicine, University of Miyazaki, 5200 Kihara, Kiyotake, Miyazaki 889-1692, Japan; Heart Center, University of Miyazaki Hospital, 5200 Kihara, Kiyotake, Miyazaki 889-1692, Japan; Heart Center, University of Miyazaki Hospital, 5200 Kihara, Kiyotake, Miyazaki 889-1692, Japan; Division of Cardiovascular Medicine and Nephrology, Department of Internal Medicine, University of Miyazaki, 5200 Kihara, Kiyotake, Miyazaki 889-1692, Japan; Division of Cardiovascular Medicine and Nephrology, Department of Internal Medicine, University of Miyazaki, 5200 Kihara, Kiyotake, Miyazaki 889-1692, Japan; Division of Cardiovascular Medicine and Nephrology, Department of Internal Medicine, University of Miyazaki, 5200 Kihara, Kiyotake, Miyazaki 889-1692, Japan; Division of Cardiovascular Medicine and Nephrology, Department of Internal Medicine, University of Miyazaki, 5200 Kihara, Kiyotake, Miyazaki 889-1692, Japan; Division of Cardiovascular Medicine and Nephrology, Department of Internal Medicine, University of Miyazaki, 5200 Kihara, Kiyotake, Miyazaki 889-1692, Japan; Department of Radiology, University of Miyazaki, 5200 Kihara, Kiyotake, Miyazaki 889-1692, Japan; Division of Orthopaedic Surgery, Department of Medicine of Sensory and Motor Organs, University of Miyazaki, 5200 Kihara, Kiyotake, Miyazaki 889-1692, Japan; Department of Cardiology, Miyazaki Medical Association Hospital, 1173, Arita, Miyazaki 880-2102, Japan; Department of Cardiology, Miyazaki Medical Association Hospital, 1173, Arita, Miyazaki 880-2102, Japan; Department of Cardiology, Miyazaki Medical Association Hospital, 1173, Arita, Miyazaki 880-2102, Japan; Division of Cardiovascular Medicine and Nephrology, Department of Internal Medicine, University of Miyazaki, 5200 Kihara, Kiyotake, Miyazaki 889-1692, Japan

**Keywords:** Longitudinal strain, Magnetic resonance image, Segmental analysis

## Abstract

**Aims:**

While the prevalence of transthyretin-derived amyloid cardiomyopathy (ATTR-CM) is on the rise, detailed understanding of its morphological and functional characteristics within the left ventricle (LV) across heart failure (HF) remains limited.

**Methods and results:**

Utilizing two-dimensional (2D) speckle-tracking echocardiography, we assessed longitudinal strain (LS) in 63 histology-confirmed ATTR-CM patients. Additionally, cardiac magnetic resonance (CMR) images measured native T1 and extracellular volume (ECV), compared with LS across 18 LV segments. Patients were categorized into three groups based on HF status: Group 1 (no HF symptoms), Group 2 (HF with preserved LV ejection fraction), and Group 3 (HF with reduced LV ejection fraction). LS analysis unveiled susceptibility to deformation in the basal inferoseptal segment, persisting even in asymptomatic cases. CMR demonstrated increasing native T1 deviation, particularly evident in segments distant from the inferoseptal region. Contrastingly, maximal ECV was consistently observed in the basal and mid-ventricular inferior-septum, even in asymptomatic individuals. Segmental LS decline correlated with ECV expansion but not with native T1 values.

**Conclusion:**

Our findings suggest that the inferoseptal segment is highly susceptible to amyloid infiltration, and 2D speckle-tracking echocardiography and CMR may serve as a valuable tool for its early detection.

## Graphical abstract

**Graphical abstract:**
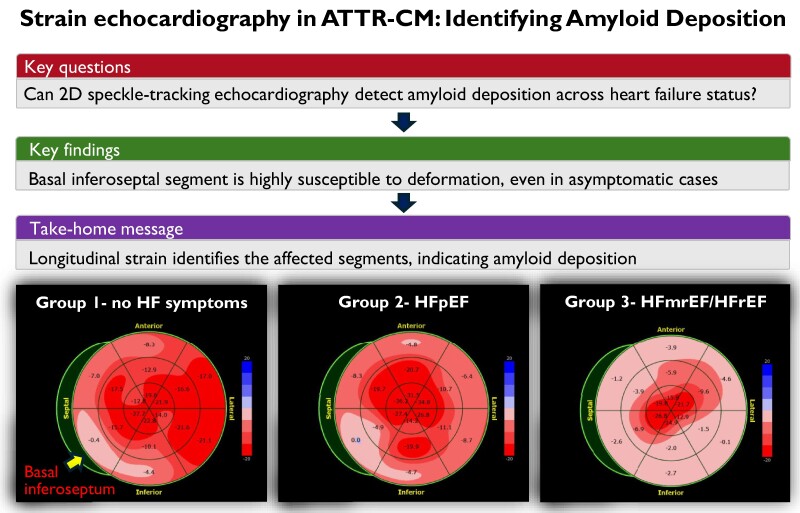

## Introduction

Systemic amyloidosis associated with cardiac involvement includes light-chain amyloidosis and transthyretin amyloidosis, the latter occurring in hereditary or wild-type forms.^1^ Cardiac involvement is characterized by hypertrophy of both ventricles. Initially, amyloid deposition in the interstitial space leads to diastolic dysfunction, with systolic function declining as deposition progresses.^1,2^

Two-dimensional (2D) speckle-tracking echocardiography involves frame-by-frame tracking of tiny echo-dense speckles within the myocardium, used to quantify global and regional myocardial deformation.^3^ The relative apical sparing pattern of longitudinal strain (LS) represents regional myocardial deformation, potentially reflecting the ubiquity of amyloid burden in the LV.^4^ This raises the hypothesis of whether amyloid fibrils initiate deposition at the basal level and progress towards the apex side. Segmental strain focuses on the analysis of strain within individual myocardial segments, valuable for identifying fine LV deformation in specific cardiomyopathies.^5–7^ This study aims to enhance our understanding of the pathogenesis of amyloid deposition in the LV. We evaluated the 18-segmental LS using 2D speckle-tracking echocardiography and compared them with the features observed in cardiac magnetic resonance (CMR) images in transthyretin-derived amyloid cardiomyopathy (ATTR-CM) patients with and without developing heart failure (HF).

## Methods

### Patients

In this retrospective, cross-sectional study, we recruited 63 patients with ATTR-CM from the University of Miyazaki Hospital between September 2018 and December 2023. ATTR-CM was confirmed by the presence of ATTR amyloid in the myocardium or synovium at the carpal tunnel on histology, along with Grades 2 and 3 cardiac uptake on ^99m^Tc-pyrophosphate (PYP) scintigraphy, in the absence of biochemical evidence of plasma cell dyscrasia.^8^ The classification of HF was defined upon hospital admission for exacerbation of HF, based on left ventricular ejection fraction (LVEF) measurements: ≤ 40% for HF with reduced LVEF (HFrEF); 41–49% for HF with mildly reduced LVEF (HFmrEF); and ≥50% for HF with preserved LVEF (HFpEF), according to the European Society of Cardiology definition.^9^ Based on clinical features, we categorized the patients into three groups: Group 1, consisted of individuals who had not yet exhibited clinical symptoms or signs of HF and did not require diuretics; Group 2 comprised patients with HFpEF; and Group 3 included those with HFmrEF/HFrEF.

Comorbidities and extracardiac manifestations of amyloidosis (e.g. carpal tunnel syndrome) were assessed via physical examination and review of medical records. Hypertension was defined as systolic blood pressure (BP) ≥ 140 mmHg, diastolic BP ≥90 mmHg, or use of antihypertensive medication. Diabetes mellitus was defined by fasting glucose ≥126 mg/dL, 2-h post-prandial glucose ≥200 mg/dL, or diabetes medication use. Dyslipidaemia was defined by total cholesterol ≥220 mg/dL, triglycerides ≥150 mg/dL, or use of dyslipidaemia medication. Severe aortic valve stenosis was defined by systolic peak velocity >4 m/s via continuous-wave Doppler ultrasound.^10^ BP was measured in a seated position at the hospital, and medication type was documented during echocardiography and CMR. Exclusion criteria included prior myocardial infarction and insufficient echocardiographic strain data. All patients were managed in accordance with the Declaration of Helsinki and provided informed consent for the anonymous publication of their data. The study protocol was approved by the University of Miyazaki Institutional Committee (Protocol number: 0-0651).

### Blood samples

Brain natriuretic peptide (BNP) levels were measured using the Abbott assay, while high-sensitive troponin T levels were measured using the Roche diagnostics assay. Estimated glomerular filtration rate (eGFR) was calculated using the standard modification of diet in renal disease study equation: eGFR (mL/min/1.73 m^2^) = 194 × (serum Cr)^−1.094^ × (age)^−0.287^ (× 0.739, when female).^11^

### Histology and immunohistochemistry

Formalin-fixed paraffin-embedded biopsies obtained from the heart or synovium were stained with Congo-red dye and/or Direct Fast Scarlet (Muto Pure Chemicals Co., Ltd., Tokyo, Japan) and observed under polarized light. The type of amyloid fibril was determined through immunohistochemical staining using a panel of antibodies against serum amyloid A protein (mouse monoclonal; DAKO), kappa_116–133_ (rabbit polyclonal), and lambda_118–134_ (rabbit polyclonal) immunoglobulin light chain, β_2_-microglobulin (rabbit polyclonal; DAKO), and transthyretin_115–124_ (rabbit polyclonal).^12^

### Genetic testing

DNA extracted from whole blood underwent amplification using a polymerase-chain-reaction assay and sequencing of exons 1–4 of the transthyretin gene to differentiate between wild-type (ATTRwt) and variant-type (ATTRv).

### Radionuclide scintigraphy

Radionuclide scintigraphy was performed 3 h post-injection of 555–740 MBq of ^99m^Tc-PYP, followed by SPECT imaging and a low-dose, non-contrast CT scan. Cardiac uptake was visually assessed using the grading scale by Perugini *et al*.^13^ (Grades 0–3), with images reviewed independently by two radiologists.

### Conventional echocardiography

Echocardiography was conducted at rest in the left lateral decubitus position using the EPIQ CVx system (Philips, Amsterdam, Netherlands) and the Vivid E95 ultrasound system (GE Healthcare, Horten, Norway), both equipped with a 1∼5-MHz transducer, by four sonographers. Standard data acquisition followed the recommendations of the American Society of Echocardiography and the European Association of Cardiovascular Imaging.^14,15^ Measurements included systolic and diastolic chamber dimensions of the LV and wall thickness obtained from 2D imaging. LVEF was determined using the modified Simpson biplane technique from two- and four-chamber orientations. Trans-mitral flow pulse-wave recordings were obtained from the apical four-chamber view to measure the peak early (*E*) diastolic velocity. The average of *E*′ septal and *E*′ lateral was assessed in the apical four-chamber view using tissue Doppler imaging, and the *E*/*e*′ ratio was calculated to evaluate diastolic function.

### 2D speckle-tracking echocardiography

We acquired apical four-, three-, and two-chamber views, which were then stored in cine-loop format. These acquisitions were triggered to the QRS complex during two heart cycles, with frame rates set between 40 and 80 frames/s. Subsequently, the cine loops underwent LS measurements using AutoStrain LV/RV/LA-automated strain measurements on the Philips EPIQ CVx system. In cases where recordings were obtained by Vivid E95, the analysis was conducted offline using vendor-independent software (TOMTEC ARENA Imaging Systems GmbA). Automated endocardial border detection for the LV was performed for each long-axis series, focusing on end-diastolic and end-systolic frames. Manual corrections were applied as needed to the automatically detected endocardial contours. The obtained strain results were expressed per segment and per level (base, mid-ventricle, and apex). For individuals with atrial fibrillation (AF), a single beat, characterized by nearly equal preceding two R–R intervals, was selected for analysis. All acquisitions and analyses were carried out by four sonographers who were blinded to the CMR results. We expressed the LS as the absolute value of the number.^16^ The relative apical longitudinal strain index (RapLSI) was calculated using the regional assessment; the mean apical LS divided by the sum of the mean basal LS and mean mid-ventricular LS.^4^ Furthermore, we employed the segmental assessment using the 18-segment model based on 2D data, represented in a bull’s eye view (see Supplementary material online, *Figure S1*).^7,16^

### CMR image acquisition

The CMR studies were conducted using a 3-T whole-body scanner (Ingenia 3.0T CX or Ingenia Elition 3.0T, Philips, Netherlands). Patients were positioned in the supine position, and imaging utilized anterior and posterior phased-array surface coils. Localisation was achieved through breath-hold real-time and steady-state free precession images, enabling accurate identification of the true anatomical axes of the heart. LV function was evaluated through cine imaging employing a segmented steady-state free precession pulse sequence. This sequence covered multiple short-axis, long-axis, and four-chamber views, encompassing the entire LV (8 mm slice thickness, breath hold, field of view 380 mm², matrix size 176 × 223, repetition time 3.0 ms, echo time 1.48 ms, flip angle 50°).

### Native T1 mapping and extracellular volume (ECV)

We employed native T1 mapping to quantify intrinsic signals from the myocardium and extracellular space, with the analysis conducted in a blinded manner to the late gadolinium enhancement (LGE) images.^17^ LGE images were acquired 10–15 min following a bolus administration of 0.1 mmol/kg gadobutrol. The acquisition, performed at mid-to-end diastole, utilized the single breath-hold shortened modified look-locker inversion recovery sequence,^18^ featuring a 10 mm slice thickness, a field of view of 380 mm², a matrix size of 144 × 142, a repetition time of 2.0 ms, an echo time of 0.95 ms, and a flip angle of 20°.

### CMR image analysis

Standard CMR analysis was conducted using commercially available software (Vitrea, VMS-001SA V8.10, Canon Medical Systems). Myocardial T1 values were measured segmentally from six equiangular segments at basal, mid-ventricular, and apical short-axis slices, ensuring avoidance of contamination from the blood pool. The data underwent analysis using IntelliSpace Portal v.10.1 (Philips). Mean native T1 and standard deviations of all pixels within each myocardial segment were documented as native T1-mean and native T1-SD, respectively.^19^ The ECV fraction, calculated based on pre-contrast and post-contrast relaxation rate measurements for the six myocardial segments in basal, mid-ventricular, and apical slices, was expressed as a percentage according to the specified formula.^20^ The institutional native T1 values, measured at the mid-ventricular septum in healthy volunteers, were 1262 ± 38 ms (Ingenia CX 3.0T, *n* = 23, male/female = 14/9, 42 ± 7 years old) and 1262 ± 27 ms (Ingenia Elition × 3.0T, *n* = 17, male/female = 11/6, 40 ± 12 years old), respectively. Due to ethical restrictions, gadobutrol administration was not permitted in these volunteers. Additionally, native T1 and ECV values at the mid-ventricular septum in another group of normal individuals were 1266 ± 30 ms and 27 ± 2% (Ingenia CX 3.0T, *n* = 20, male/female = 15/5, 61 ± 16 years old).

### Statistical analysis

Normality was assessed via the Shapiro–Wilk test. Continuous data were reported as mean ± standard deviation or median (25th–75th percentiles), and categorical variables as counts (percentages). Categorical variables among three groups were compared using Pearson’s *χ*^2^ test. For multiple comparisons, one-way ANOVA (parametric) or Kruskal–Wallis (non-parametric) tests with Tukey *post hoc* analysis were applied. Pearson correlation (*r*) was used to assess the linear relationship between LS by echocardiogram and MRI-derived parameters (native T1 and ECV) across 18 myocardial segments, with the coefficient of determination (*r*²) quantifying variance explained. The intraclass correlation coefficient evaluated the reproducibility of 2D speckle-tracking echocardiography among four sonographers. Statistical significance was set at *P* < 0.05. Analyses and graphs were generated using SPSS version 29 (IBM Corporation, Armonk, New York, USA) and GraphPad Prism 8.0 (La Jolla, California, USA).

## Results

### Patient population

**Figure 1:**
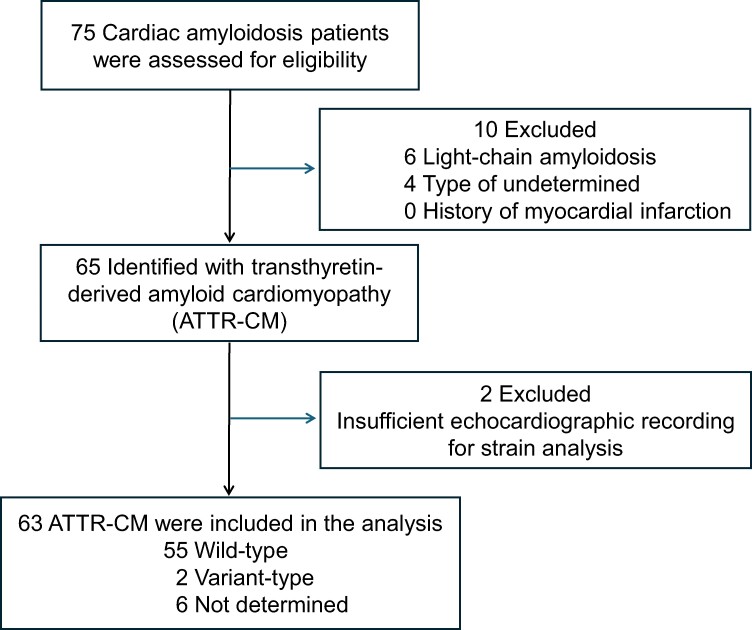
Patient recruitment flowchart.

*Figure 1* presented a flowchart depicting the recruitment process and excluded patients, and we analysed 63 ATTR-CM patients in this study. *Table 1* presents the clinical data of the study participants. ATTR-CM predominantly affected older males, with 68% having hypertension, 22% having diabetes mellitus, and 32% having dyslipidaemia. The diagnosis was confirmed histologically with heart specimens (*n* = 61) and synovium specimens (*n* = 2). Genetic testing revealed wild-type ATTR in 87%, variant-type ATTR in 3%, and 10% were not tested. In group analysis, Group 3 exhibited higher levels of BNP and troponin T, along with lower eGFR compared to either Group 1 or 2. Loop diuretics, mineral corticoid receptor antagonists, beta blockers, and sodium glucose transporter 2 inhibitor were more frequently prescribed in Group 3 than in Group 1 or 2.

**Table 1:** Baseline characteristics of the study population categorized by clinical groups

	All patients (*n* = 63)	Group 1 (*n* = 10)	Group 2 (*n* = 17)	Group 3 (*n* = 36)	*P*-value
Age at diagnosis (years)	78 [72, 81]	74 [70, 79]	78 [71, 83]	78 [73, 80]	0.459
Male sex, *n* (%)	59 (94)	8 (89)	16 (94)	35 (95)	0.874
Body mass index, kg/m^2^	23 [21, 24]	23 [22, 25]	23 [21, 24]	23 [21, 25]	0.754
Comorbidities					
Hypertension, *n* (%)	43 (68)	9 (90)	9 (53)	25 (69)	0.132
Diabetes mellitus, *n* (%)	14 (22)	1 (11)	5 (29)	8 (22)	0.503
Dyslipidaemia, *n* (%)	20 (32)	5 (56)	6 (35)	9 (24)	0.302
Coronary interventions, *n* (%)	12 (19)	0 (0)	5 (29)	7 (19)	0.170
Severe aortic valve stenosis, *n* (%)	2 (3)	0 (0)	1 (6)	1 (3)	0.687
AF/flutter, *n* (%)	25 (40)	1 (11)	9 (53)	15 (41)	0.083
Carpal tunnel syndrome, *n* (%)	32 (51)	6 (60)	8 (47)	18 (50)	0.801
SBP (mmHg)	122 ± 18	133 ± 13	119 ± 18	120 ± 18	0.086
DBP (mmHg)	71 ± 13	82 ± 8	70 ± 17	69 ± 11*	0.025
Pulse rate (b.p.m.)	72 ± 13	77 ± 16	73 ± 10	70 ± 14	0.340
Biopsy sites					0.212
Heart	61	9	16	36	
Synovium	2	1	1	0	
^99m^Tc-PYP (grade≧2), *n* (%)	60/60 (100)	8/8 (100)	17/17 (100)	35/35 (100)	0.629
ATTR type					0.134
Wild, *n* (%)	55 (87)	9 (90)	17 (100)	29 (80)	
Variant, *n* (%)	2 (3)	1 (10)	0 (0)	1 (3)	
Not tested, *n* (%)	6 (10)	0 (0)	0 (0)	6 (17)	
Biomarkers					
BNP (pg/mL)	203 [103, 295]	75 [36, 109]	203 [111, 295]**	252 [153, 338]**	<0.001
Troponin T (ng/mL)	0.05 [0.03, 0.07]	0.03 [0.02, 0.04]	0.05 [0.03, 0.06]	0.06 [0.04, 0.08]** (*n* = 35)	0.008
eGFR (mL/min/1.73 m^2^)	53 ± 15	64 ± 13	52 ± 16	50 ± 14*	0.036
Medications					
Loop diuretics, *n* (%)	43 (68)	0 (0)	13 (76)	30 (81)	<0.001
ACEi/ARBs, *n* (%)	35 (56)	7 (70)	7 (41)	21 (58)	0.304
MRAs, *n* (%)	33 (52)	1 (10)	10 (59)	22 (61)	0.014
Beta blockers, *n* (%)	25 (40)	0 (0)	8 (47)	17 (46)	0.020
ARNI, *n* (%)	5 (8)	0 (0)	2 (12)	3 (8)	0.546
SGLT2i, *n* (%)	23 (37)	0 (0)	7 (41)	16 (43)	0.032
sGC stimulator, *n* (%)	2 (3)	0 (0)	1 (6)	1 (3)	0.687
PPM, *n* (%)	1 (2)	1 (10)	0 (0)	0 (0)	0.068
CRT-D, *n* (%)	1 (2)	0 (0)	0 (0)	1 (3)	0.683

Continuous variables were expressed as mean ± standard deviation or median [25%, 75% quantile], and categorical variables were expressed as absolute numbers (percentage). One-way ANOVA or Kruskal–Wallis was performed for multiple comparisons, and Tukey adjustment was applied after the overall three groups. Categorical variables in three groups were assessed with the Pearson’s *χ*^2^ test.

AF, atrial fibrillation; SBP, systolic blood pressure; DBP, diastolic blood pressure; ^99m^Tc-PYP, ^99m^Tc-phyrophosphate; ATTR, transthyretin; BNP, brain natriuretic peptide; eGFR, estimated glomerular filtration rate; ACEi, angiotensin converting enzyme inhibitor; ARB, angiotensin II type 1 receptor blocker; MRA, mineral corticoid receptor antagonist; ARNI, angiotensin receptor neprilysin inhibitor; SGLT2i, sodium-glucose cotransporter 2 inhibitor; sGC, sodium guanylate cyclase; PPM, permanent pacemaker; CRT-D, cardiac resynchronisation therapy-defibrillation.

^*^ *P* < 0.05, **P < 0.01 vs. Group 1; ^#^*P* < 0.05, ^##^*P* < 0.01 vs. Group 2.

### Echocardiographic measurements

In *Table 2*, Group 3 exhibited increased thickness in the LV septal and posterior walls compared to Group 1, while also showing a lower LVEF than either Group 1 or 2. Additionally, Group 3 demonstrated an increased ratio of *E* to *e*′ compared to Group 1. In 2D speckle-tracking echocardiography, Group 3 had a lower global LS than Group 1 or 2. Regional LS was predominantly reduced at the base level, compared to the mid-ventricle and apex levels. The value further decreased in the base and mid-ventricle in Group 2 and/or 3 compared to Group 1. In the apex, Group 2 exhibited higher LS than Group 1, while Group 3 showed a lower value than Group 2. RapLSI (>1) was observed in 79% of all patients, with a higher frequency in Group 3 compared to Group 1 or Group 2.

**Table 2:** Echocardiographic and CMR parameters in study groups

	All patients	Group 1	Group 2	Group 3	*P*-value
**Echocardiogram**	(*n* = 63)	(*n* = 10)	(*n* = 17)	(*n* = 36)	
IVSTd (mm)	17 [15, 18]	15 [12, 17]	17 [15, 17]	17 [16, 18]*	0.043
LVPWTd (mm)	16 ± 2	13 ± 3	15 ± 2	16 ± 2**	0.004
LVEF (%)	47 ± 10	52 ± 9	57 ± 5	40 ± 7*^##^	<0.001
***E***/***e***′	17 ± 5	13 ± 5	16 ± 3	19 ± 6**	0.009
Global LS (%)	11 [8, 13]	14 [11, 16]	14 [12, 16]	9 [7, 11]^**##^	<0.001
Regional LS (%)					
Base	5 [3, 8]	10 [7, 12]	7 [5, 9]*	4 [3, 6]^**#^	<0.001
Mid-ventricle	8 [5, 10]	11 [8, 15]	10 [9, 14]	6 [4, 8]^**##^	0.002
Apex	18 ± 6	19 ± 6	24 ± 5*	16 ± 3^##^	<0.001
RapLSI	1.33 [1.02, 1.78]	0.92 [0.71, 1.72]	1.38 [1.02, 1.78]*	1.38 [1.09, 1.87]**	0.003
RapLSI (>1), ***n*** (%)	50 (79)	4 (40)	14 (82)	32 (89)	0.003
**CMR**	(*n* = 48)	(*n* = 9)	(*n* = 14)	(*n* = 25)	
LVEF (%)	53 ± 12	55 ± 9	63 ± 10	47 ± 11^##^	<0.001
CI (L/min/m^2^)	2.1 ± 0.6	1.9 ± 0.7	2.1 ± 0.5	2.2 ± 0.6	0.580
LVEDV (mL/m^2^)	63 ± 18	55 ± 16	54 ± 12	72 ± 17*^##^	0.001
LVESV (mL/m^2^)	27 [21, 41]	24 [20, 28]	21 [14, 27]	40 [27, 47]^**##^	<0.001
Regional native T1 (ms)	(*n* = 47)	(*n* = 9)	(*n* = 14)	(*n* = 24)	
Base	1441 ± 53	1392 ± 46	1457 ± 40**	1450 ± 53*	0.006
Mid-ventricle	1418 [1386, 1465]	1368 [1293, 1340]	1431 [1389, 1460]	1427 [1409, 1494]**	0.010
Apex	1434 ± 97	1388 ± 109	1456 ± 64	1439 ± 86	0.250
Regional ECV (%)	(*n* = 41)	(*n* = 9)	(*n* = 10)	(*n* = 22)	
Base	56 [46, 61]	46 [39, 51]	52 [48, 60]	61 [54, 62]^**#^	<0.001
Mid-ventricle	49 [44, 52]	43 [32, 45]	48 [42, 51]	51 [49, 55]^**#^	0.004
Apex	45 ± 8	39 ± 6	42 ± 9	48 ± 5**	0.003

Continuous variables were expressed as mean ± standard deviation or median [25%, 75% quantile], and categorical variables were expressed as absolute numbers (percentage). One-way ANOVA or Kruskal–Wallis was performed for multiple comparisons, and Tukey adjustment was applied after the overall three groups. Categorical variables in three groups were assessed with the Pearson’s *χ*^2^ test. LS was expressed as the absolute value of the number.

IVSTd, interventricular septal thickness at end-diastole; LVPWTd, left ventricular posterior wall thickness at end-diastole; RapLSI, relative apical longitudinal strain index; LVEF, left ventricular ejection fraction; LS, longitudinal strain; CI, cardiac index; LVEDV, left ventricular end-diastolic volume; LVESV, left ventricular end-systolic volume; ECV, extracellular volume.

^*^ *P* < 0.05, **P < 0.01 vs. Group 1; ^#^*P* < 0.05, ^##^*P* < 0.01 vs. Group 2.

### LV segmental strain in subgroups

**Figure 2:**
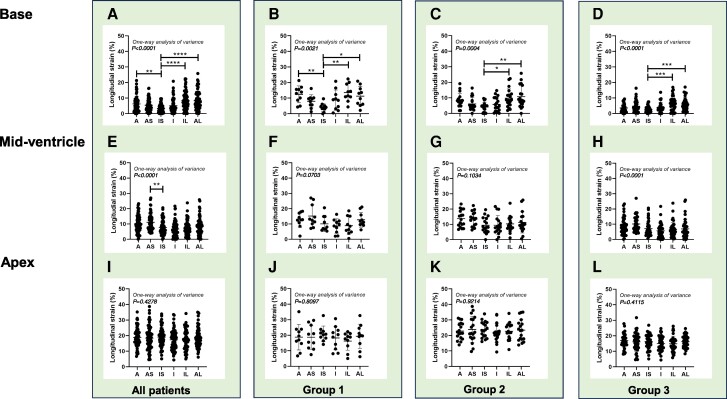
Longitudinal strain (LS) for each of the six myocardial segments in the base (*A–D*), mid-ventricle (*E–H*), and apex (*I–L*) slices across all patients (*n* = 63; *A*, *E*, *I*), Group 1 (*n* = 10; *B*, *F, J*), Group 2 (*n* = 17; *C*, *G, K*), and Group 3 (*n* = 36; *D*, *H, L*). *P < 0.05, **P < 0.01, ***P < 0.001, ****P < 0.0001 vs. IS, assessed by one-way ANOVA followed by Tukey’s test. A, anterior; AS, anteroseptal; IS, inferoseptal; I, inferior; IL, inferolateral; AL, anterolateral. LS was expressed as the absolute value of the number.

**Figure 3:**
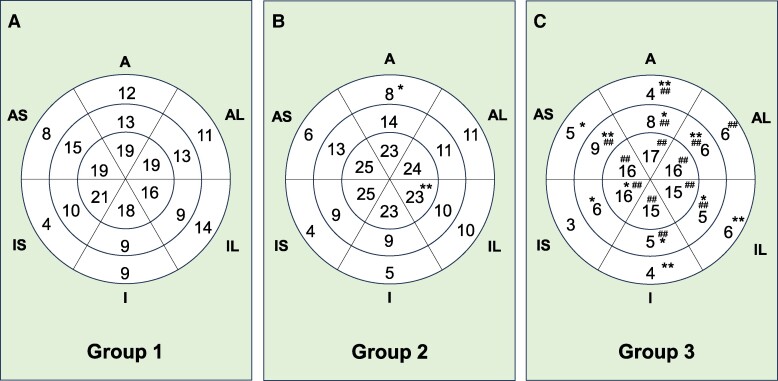
Between-group analysis of longitudinal strain (LS) in the 18 myocardial segments. The average LS is indicated in the centre of each plot. *P < 0.05, **P < 0.01 vs. Group 1; ^##^*P* < 0.01 vs. Group 2, assessed by one-way ANOVA followed by Tukey’s test. (*A*, *n* = 10; *B*, *n* = 17; *C*, *n* = 36). A, anterior; AS, anteroseptal; IS, inferoseptal; I, inferior; IL, inferolateral; AL, anterolateral. LS was expressed as the absolute value of the number.

*Figure 2A–D* illustrate that the basal inferoseptal segment consistently exhibited the lowest LS value among the six segments, irrespective of their HF status. In the mid-ventricle (*Figure 2E–H*), LS at the inferoseptal segment was significantly lower than the anteroseptal segment across all patients. In the apex (*Figure 2I–L*), however, the degree of LS did not differ significantly among the six segments. *Figure 3A–C* depict the average LS values in the respective segments. Group 2 exhibited a significantly lower value in the basal anterior segment but a higher value in the apex of the inferolateral segment compared to Group 1. Group 3 had the lowest LS values across all basal, mid-ventricle, and apex segments, in comparison to Group 1 and/or Group 2. In 13 cases where the RapLSI was <1, we found that LS at the basal inferoseptal segment was considerably impaired, exhibiting a median value of 4.7% (25th percentile: 2.8%, 75th percentile: 6.5%). Graphical abstract illustrates examples of the LS bull’s eye plot in their respective groups.

### Reproducibility of 2D speckle-tracking echocardiographic measurements

In 19 consecutive ATTR-CM patients, the intraclass correlation coefficient consistently exceeded 0.85 for every segment across the basal, mid-ventricle, and apex slices in the LV (see Supplementary material online, *Table S1*).

### CMR assessments

**Figure 4:**
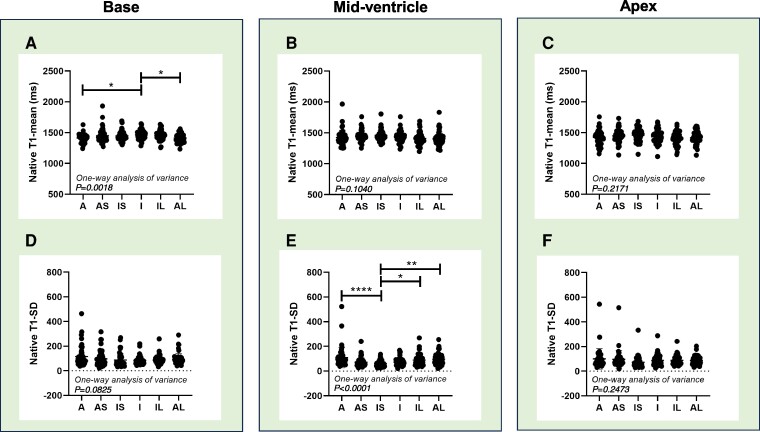
Native T1 mean value (*A–C*) and standard deviation of native T1 (*D–F*) for each of the six myocardial segments in the base (*A*, *D*), mid-ventricle (*B*, *E*), and apex (*C*, *F*) slices across all patients (*n* = 47). *P < 0.05, **P < 0.01, ****P < 0.0001 vs. IS or I, assessed by one-way ANOVA followed by Tukey’s test. A, anterior; AS, anteroseptal; IS, inferoseptal; I, inferior; IL, inferolateral; AL, anterolateral.

**Figure 5:**
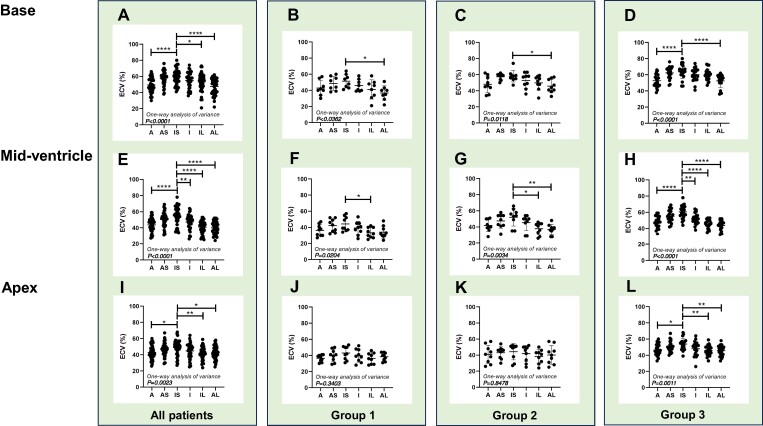
Extracellular volume (ECV) for each of the six myocardial segments in the base (*A–D*), mid-ventricle (*E–H*), and apex (*I–L*) slices across all patients (*n* = 41; *A, E, I*), Group 1 (*n* = 9; *B, F, J*), Group 2 (*n* = 10; *C*, *G*, *K*), and Group 3 (*n* = 22; *D, H, L*). *P < 0.05, **P < 0.01, ***P < 0.001, ****P < 0.0001 vs. IS, assessed by one-way ANOVA followed by Tukey’s test. A, anterior; AS, anteroseptal; IS, inferoseptal; I, inferior; IL, inferolateral; AL, anterolateral.

Cine-CMR was conducted on 48 patients, and *Table 2* reveals that cardiac index was consistent across the three groups, with Group 3 exhibiting increased LV end-diastolic and end-systolic volumes compared to Groups 1 and 2. Native T1 mapping was performed in 47 patients. Groups 2 and 3 displayed higher mean T1 values than Group 1 at the base, and Group 3 had a higher value than Group 1 at the mid-ventricle slice (*Table 2*). *Figure 4A–C* depict a continuous increase in segmental native T1-mean values in the base, mid-ventricle, and apex slices. *Figure 4D–F* illustrate that the inferior (base) and inferoseptal segments (mid-ventricle) had lower native T1 deviation than other segments, respectively. Contrast images were utilized for 41 patients, and as indicated in *Table 2*, the basal slice had a higher average ECV value than the mid-ventricle and apex slices, with Group 3 showing a higher value than Groups 1 and 2 in the base, mid-ventricle, and apex slices. *Figure 5* illustrates that the inferoseptal segment consistently exhibited the highest ECV value across the base, mid-ventricle, and apex in all patients, with this pattern persisting in the basal and mid-ventricular slices in Groups 1–3. In contrast, analyses in healthy volunteers (see Supplementary material online, *Figure S2*) and another group of normal individuals (see Supplementary material online, *Figure S3*) from our institution showed a slight increase in native T1 and ECV predominantly in the anteroseptal segment. We also compared native T1 and ECV values across segments in two patients with ATTRv (1, heterozygous c.424G>A p.Val142Ile; 1, heterozygous c.148G>A p.Val50Met) with those in patients with ATTRwt, finding substantial overlap in native T1 values, though ECV values were generally lower in ATTRv (see Supplementary material online, *Tables S2* and *S3*).

### Correlation between echocardiographic segmental LS assessments and CMR data

**Figure 6:**
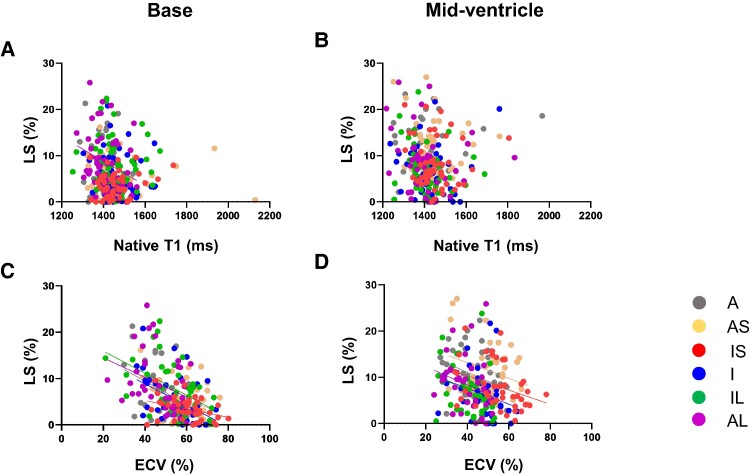
Correlation between longitudinal strain (LS) assessed by 2D speckle-tracking echocardiography and native T1 (*A*, *B*) and extracellular volume (ECV) (*C*, *D*) assessed by cardiac magnetic resonance imaging in the six segments of the basal and mid-ventricular slices. A, anterior; AS, anteroseptal; IS, inferoseptal; I, inferior; IL, inferolateral; AL, anterolateral. LS was expressed as the absolute value of the number.

*Figure 6A* and *B* illustrate that segmental LS did not show a correlation with native T1 values at the corresponding sites in the basal, mid-ventricle, and apex slices, except for the basal, anterolateral segment (*r* = −0.294, *r*^2^ = 0.086, *P* = 0.045). *Figure 6C* illustrates that LS correlated with ECV at the corresponding segments of the basal slice (*anterior*, *r* = −0.427, *r*^2^ = 0.182, *P* = 0.005; *anteroseptal*, *r* = −0.314, *r*^2^ = 0.099, *P* = 0.045; *inferoseptal*, *r* = −0.328, *r*^2^ = 0.108, *P* = 0.036; *inferior*, *r* = −0.468, *r*^2^ = 0.219, *P* = 0.002; *inferolateral*, *r* = −0.485, *r*^2^ = 0.235, *P* = 0.001; *anterolateral*, *r* = −0.336, *r*^2^ = 0.113, *P* = 0.032). *Figure 6D* illustrates their significant relationship at the mid-ventricle slices (*anterior*, *r* = −0.305, *r*^2^ = 0.093, *P* = 0.053; *anteroseptal*, *r* = −0.270, *r*^2^ = 0.0729, *P* = 0.088; *inferoseptal*, *r* = −0.364, *r*^2^ = 0.132, *P* = 0.019; *inferior*, *r* = −0.342, *r*^2^ = 0.117, *P* = 0.029; *inferolateral*, *r* = −0.366, *r*^2^ = 0.134, *P* = 0.019; *anterolateral*, *r* = −0.335, *r*^2^ = 0.112, *P* = 0.032). However, the relationship was not detected at the apical slice (data not shown).

## Discussion

The primary objective of this study was to characterize segmental LS across the clinical spectrum of ATTR-CM patients using 2D speckle-tracking echocardiography. We report for the first time that segmental LS displays unique characteristics, with the highest deterioration in the inferoseptal segment of basal slices, and to a lesser extent in other segments. Importantly, this trend persisted in patients who have not yet exhibited symptoms or signs of HF.

Amyloid fibrils initially distribute at the subendocardial layer and progress to cover the entire transmural circumference.^21,22^ We chose 2D speckle-tracking to detect the endocardial border of the LV contour, where the internal oblique myofibers responsible for endocardial LS are located. The deposition of amyloid fibrils at the basal septum leads to heterogeneously increased LV stiffness,^23^ potentially enhancing regional LS in the apex of patients with HF with preserved ejection fraction (HFpEF—Group 2). This characteristic may be specific to ATTR-CM; a study demonstrated uniformly reduced regional LS in patients with HFpEF.^24^ However, in patients with HF with mildly reduced or reduced ejection fraction (HFmrHF/HFrEF—Group 3), regional LS in the apex decreased. This decrease was compensated by an increase in LV chamber volume, contributing to the maintenance of the cardiac index.

To better understand segmental LV deformity, we assessed CMR data and compared them with echocardiographic data in a Bull’s eye plot. Native T1 values demonstrated equivalent increases, whereas the ECV at the inferoseptal segment exhibited the highest value among the six segments, aligning with the impairment of segmental LS. The weak correlation between LS and ECV suggests that amyloid deposition alone may not directly influence LS; instead, other factors constituting the extracellular space, such as fibrosis, oedema, and microvascular changes, might also contribute.^25,26^ Importantly, microvascular dysfunction has a significant impact on LS.^27,28^ The median time interval between these two imaging modalities was 52 days (25th percentile: 29 days; 75th percentile: 122 days), which could also have influenced the observed inverse correlation.

CMR data suggest minimal myocardial tissue inhomogeneity, as assessed by native T1-SD, in the inferior and inferoseptal segments, with increased inhomogeneity observed in more distant regions, particularly in the basal and mid-ventricular slices. Our findings are supported by a prior study indicating that the basilar ventricular septum is site of the most prominent amyloid deposition in early cases of a forensic autopsy-based series.^29^ The vulnerability of basal inferoseptal LS to deterioration in ATTR-CM patients remains unclear. Interestingly, in healthy adult volunteers, the basal anteroseptal segment exhibited the lowest LS (18.7%), with an average value of 20.8% across six segments.^30^ This aligns with segmental ECV analysis in normal individuals from our institution, which shows a slight increase in the basal anteroseptal segment (see Supplementary material online, *Figure S3G*). Further studies are needed to explore the mechanisms behind preferential amyloid deposition in the inferoseptal segment of the LV.

This study included 40% patients with AF/atrial flutter. AF can significantly impact the results of LS: greatly increased beat-to-beat duration variability, and absence of atrial contraction, leading to impaired global LS in AF compared to sinus rhythm.^31^ This increases the complexity of strain analysis in AF patients and may require the use of advanced algorithms or manual adjustments to obtain reliable data.^32^ Despite these challenges, LS remains a valuable tool for assessing segmental deformation in patients with ATTR-CM.

## Study limitations

Acknowledging the potential for selection bias that may have influenced the results is essential. For instance, we encountered two cases (3%) exhibiting severe aortic valve stenosis. However, it is worth noting that ATTR-CM patients co-present in 4–16% of cases of severe aortic valve stenosis.^33^ Two patients had a permanent pacemaker implantation, and a cardiac resynchronisation therapy-defibrillation implantation, respectively. Right ventricular pacing has reduced LS at all segments in the LV apex, as well as at the inferior and septal segments in the mid-LV.^34^ Seven patients were unable to undergo assessment of contrast CMR images due to renal impairment. Genetic testing plays a crucial role in the evaluation of ATTR amyloidosis.^35^ In our study, six patients did not undergo genetic testing, particularly those who did not receive any disease-modifying therapies.

## Conclusions

Our study highlights the pronounced vulnerability of the inferoseptal segment to deformation in the clinical spectrum of ATTR-CM, potentially reflecting the extent of amyloid deposition and other extracellular constituent. Moreover, our findings suggest that both LS by 2D speckle-tracking echocardiography and ECV by CMR seem to identify this segment as particularly affected.

## Supplementary Material

oeae076_Supplementary_Data

## Data Availability

The original contributions presented in this study are detailed in the article and Supplementary materials. For additional inquiries, please contact the corresponding author.
