# Supplementary material for: Basal inferoseptal segment is highly susceptible to deformation in the clinical spectrum of transthyretin-derived amyloid cardiomyopathy
Source: Eur Heart J Open. 2024 Sep 2;4(5):oeae076. doi: 10.1093/ehjopen/oeae076 (PMC11404357; doi:10.1093/ehjopen/oeae076)
Supplement: oeae076_Supplementary_Data [file oeae076_supplementary_data.zip › Supplemental Figure 1 Tsuruda T et al..pptx]

## Slide 1
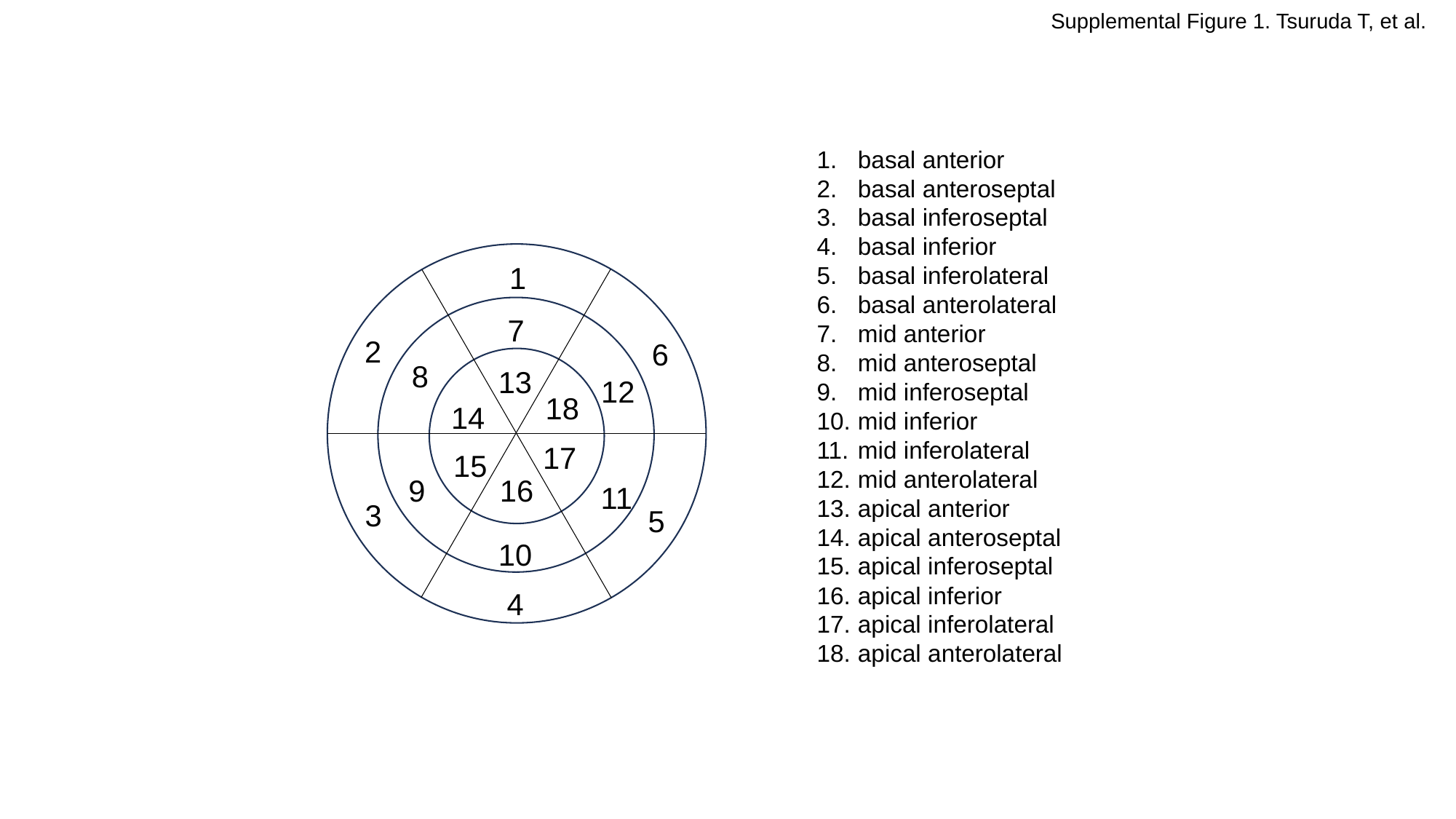

Supplemental Figure 1. Tsuruda T, et al.
basal anterior
basal anteroseptal
basal inferoseptal
basal inferior
basal inferolateral
basal anterolateral
mid anterior
mid anteroseptal
mid inferoseptal
mid inferior
mid inferolateral
mid anterolateral
apical anterior
apical anteroseptal
apical inferoseptal
apical inferior
apical inferolateral
apical anterolateral
1
7
2
6
8
13
12
18
14
17
15
9
16
11
3
5
10
4
