# Supplementary material for: Basal inferoseptal segment is highly susceptible to deformation in the clinical spectrum of transthyretin-derived amyloid cardiomyopathy
Source: Eur Heart J Open. 2024 Sep 2;4(5):oeae076. doi: 10.1093/ehjopen/oeae076 (PMC11404357; doi:10.1093/ehjopen/oeae076)
Supplement: oeae076_Supplementary_Data [file oeae076_supplementary_data.zip › Supplemental Table 1 Tsuruda T et al..pptx]

## Slide 1
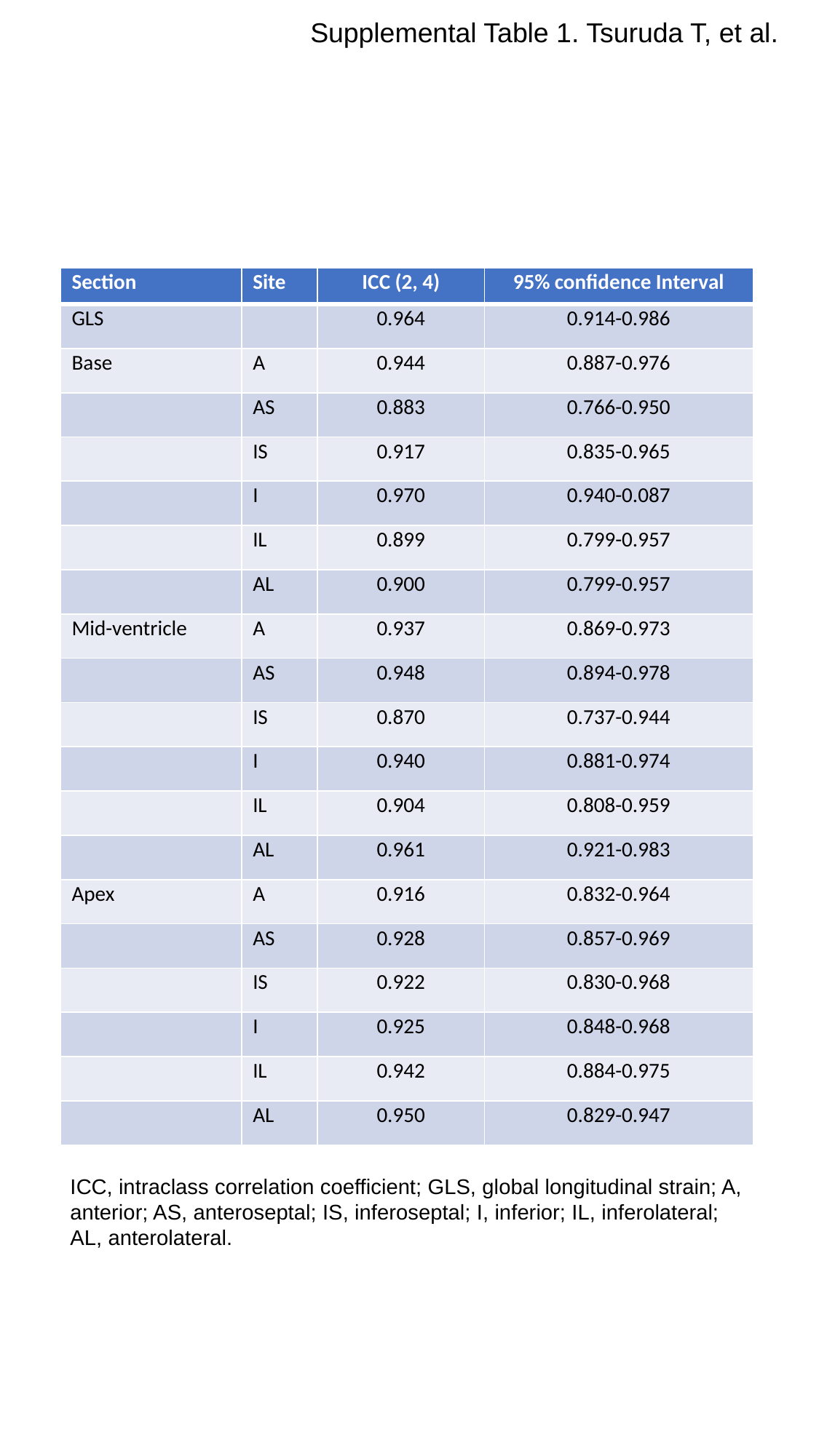

Supplemental Table 1. Tsuruda T, et al.
| Section | Site | ICC (2, 4) | 95% confidence Interval |
| --- | --- | --- | --- |
| GLS | | 0.964 | 0.914-0.986 |
| Base | A | 0.944 | 0.887-0.976 |
| | AS | 0.883 | 0.766-0.950 |
| | IS | 0.917 | 0.835-0.965 |
| | I | 0.970 | 0.940-0.087 |
| | IL | 0.899 | 0.799-0.957 |
| | AL | 0.900 | 0.799-0.957 |
| Mid-ventricle | A | 0.937 | 0.869-0.973 |
| | AS | 0.948 | 0.894-0.978 |
| | IS | 0.870 | 0.737-0.944 |
| | I | 0.940 | 0.881-0.974 |
| | IL | 0.904 | 0.808-0.959 |
| | AL | 0.961 | 0.921-0.983 |
| Apex | A | 0.916 | 0.832-0.964 |
| | AS | 0.928 | 0.857-0.969 |
| | IS | 0.922 | 0.830-0.968 |
| | I | 0.925 | 0.848-0.968 |
| | IL | 0.942 | 0.884-0.975 |
| | AL | 0.950 | 0.829-0.947 |
ICC, intraclass correlation coefficient; GLS, global longitudinal strain; A, anterior; AS, anteroseptal; IS, inferoseptal; I, inferior; IL, inferolateral; AL, anterolateral.
