# Supplementary material for: Basal inferoseptal segment is highly susceptible to deformation in the clinical spectrum of transthyretin-derived amyloid cardiomyopathy
Source: Eur Heart J Open. 2024 Sep 2;4(5):oeae076. doi: 10.1093/ehjopen/oeae076 (PMC11404357; doi:10.1093/ehjopen/oeae076)
Supplement: oeae076_Supplementary_Data [file oeae076_supplementary_data.zip › Supplemental Table 2 Tsuruda T et al..pptx]

## Slide 1
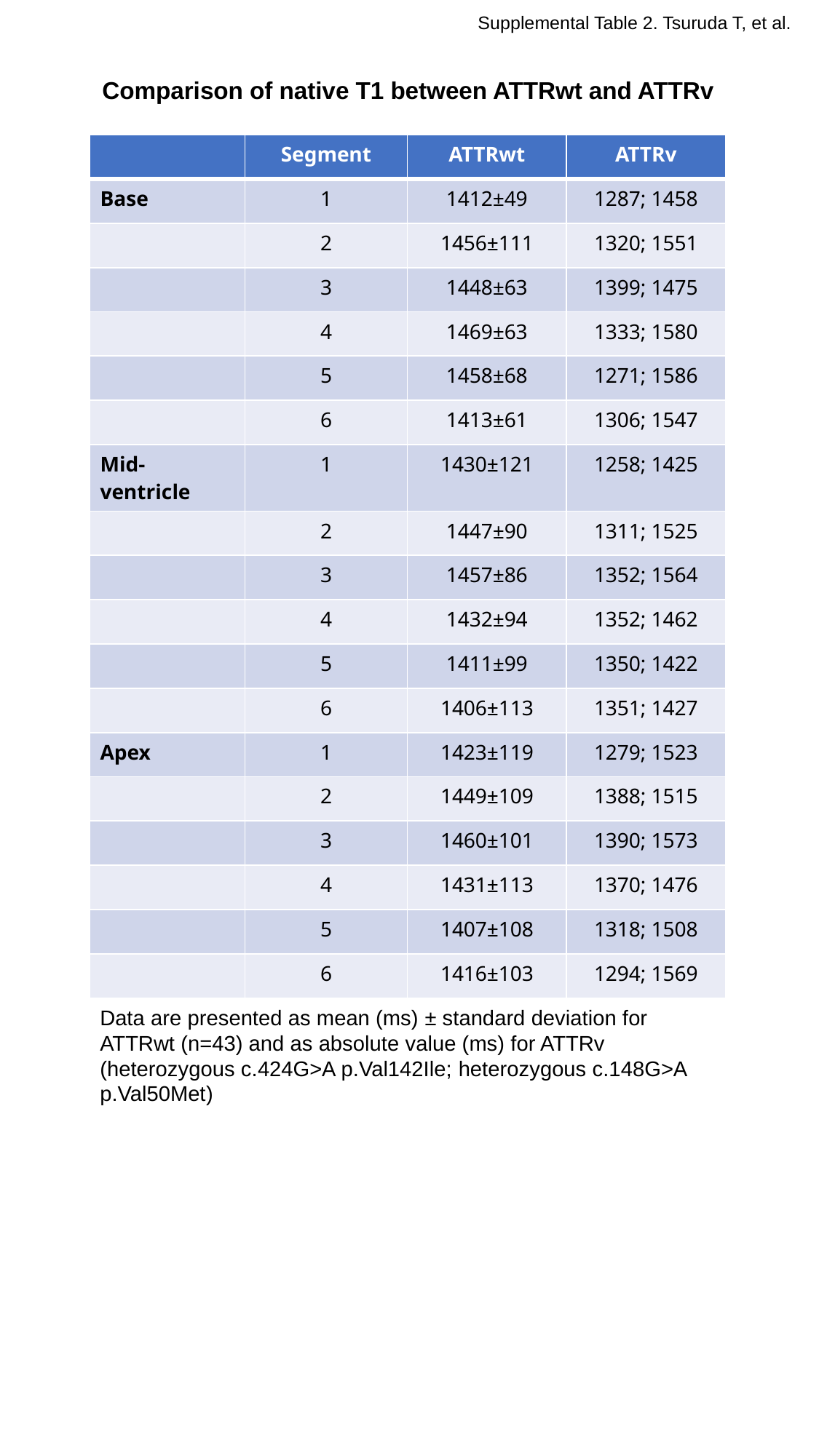

Supplemental Table 2. Tsuruda T, et al.
Comparison of native T1 between ATTRwt and ATTRv
| | Segment | ATTRwt | ATTRv |
| --- | --- | --- | --- |
| Base | 1 | 1412±49 | 1287; 1458 |
| | 2 | 1456±111 | 1320; 1551 |
| | 3 | 1448±63 | 1399; 1475 |
| | 4 | 1469±63 | 1333; 1580 |
| | 5 | 1458±68 | 1271; 1586 |
| | 6 | 1413±61 | 1306; 1547 |
| Mid-ventricle | 1 | 1430±121 | 1258; 1425 |
| | 2 | 1447±90 | 1311; 1525 |
| | 3 | 1457±86 | 1352; 1564 |
| | 4 | 1432±94 | 1352; 1462 |
| | 5 | 1411±99 | 1350; 1422 |
| | 6 | 1406±113 | 1351; 1427 |
| Apex | 1 | 1423±119 | 1279; 1523 |
| | 2 | 1449±109 | 1388; 1515 |
| | 3 | 1460±101 | 1390; 1573 |
| | 4 | 1431±113 | 1370; 1476 |
| | 5 | 1407±108 | 1318; 1508 |
| | 6 | 1416±103 | 1294; 1569 |
Data are presented as mean (ms) ± standard deviation for ATTRwt (n=43) and as absolute value (ms) for ATTRv (heterozygous c.424G>A p.Val142Ile; heterozygous c.148G>A p.Val50Met)
