# Supplementary material for: Basal inferoseptal segment is highly susceptible to deformation in the clinical spectrum of transthyretin-derived amyloid cardiomyopathy
Source: Eur Heart J Open. 2024 Sep 2;4(5):oeae076. doi: 10.1093/ehjopen/oeae076 (PMC11404357; doi:10.1093/ehjopen/oeae076)
Supplement: oeae076_Supplementary_Data [file oeae076_supplementary_data.zip › Supplemental Table 3 Tsuruda T et al..pptx]

## Slide 1
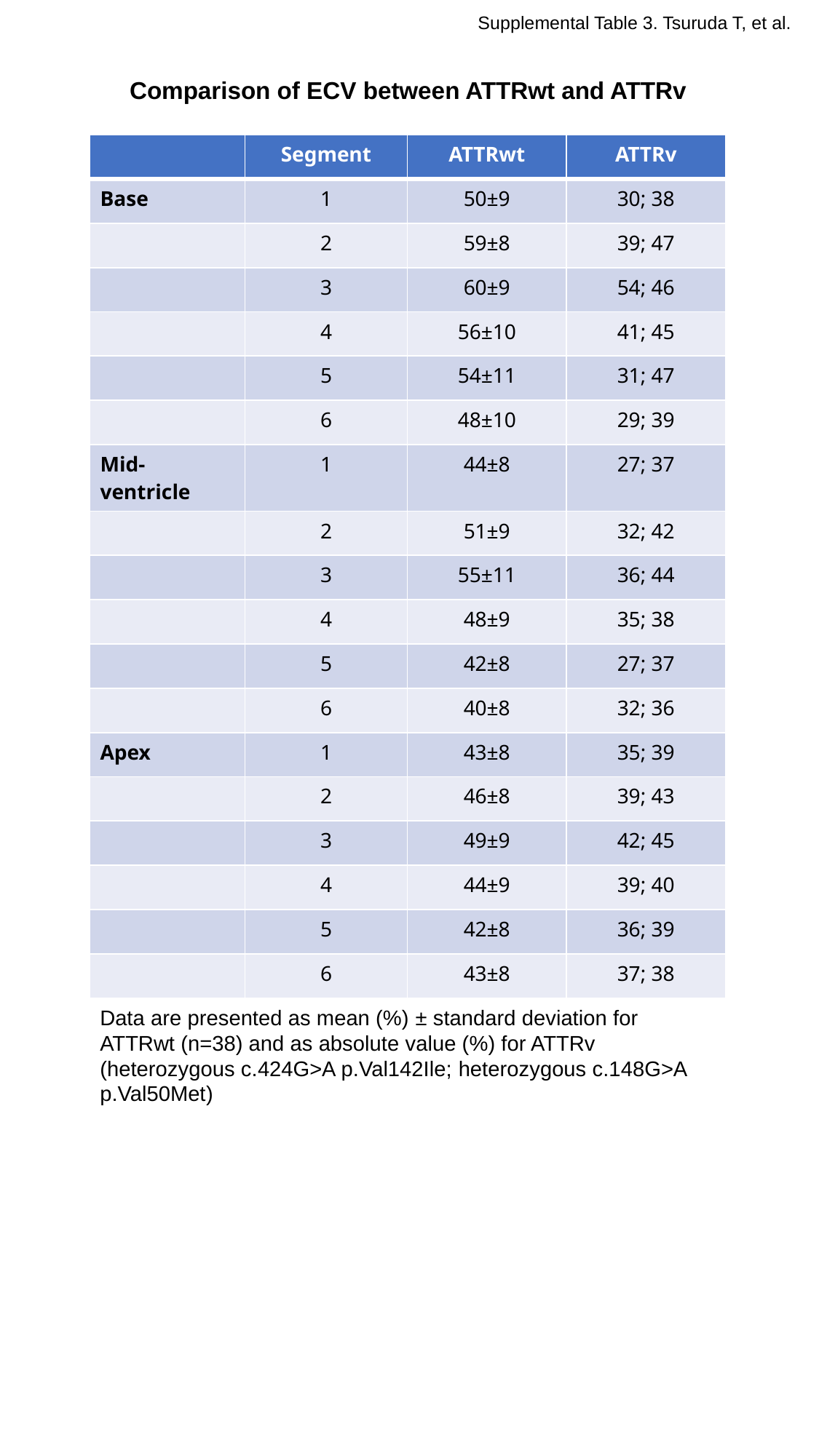

Supplemental Table 3. Tsuruda T, et al.
Comparison of ECV between ATTRwt and ATTRv
| | Segment | ATTRwt | ATTRv |
| --- | --- | --- | --- |
| Base | 1 | 50±9 | 30; 38 |
| | 2 | 59±8 | 39; 47 |
| | 3 | 60±9 | 54; 46 |
| | 4 | 56±10 | 41; 45 |
| | 5 | 54±11 | 31; 47 |
| | 6 | 48±10 | 29; 39 |
| Mid-ventricle | 1 | 44±8 | 27; 37 |
| | 2 | 51±9 | 32; 42 |
| | 3 | 55±11 | 36; 44 |
| | 4 | 48±9 | 35; 38 |
| | 5 | 42±8 | 27; 37 |
| | 6 | 40±8 | 32; 36 |
| Apex | 1 | 43±8 | 35; 39 |
| | 2 | 46±8 | 39; 43 |
| | 3 | 49±9 | 42; 45 |
| | 4 | 44±9 | 39; 40 |
| | 5 | 42±8 | 36; 39 |
| | 6 | 43±8 | 37; 38 |
Data are presented as mean (%) ± standard deviation for ATTRwt (n=38) and as absolute value (%) for ATTRv (heterozygous c.424G>A p.Val142Ile; heterozygous c.148G>A p.Val50Met)
