# Supplementary figures and images for: Basal inferoseptal segment is highly susceptible to deformation in the clinical spectrum of transthyretin-derived amyloid cardiomyopathy
Source: Eur Heart J Open. 2024 Sep 2;4(5):oeae076. doi: 10.1093/ehjopen/oeae076 (PMC11404357; doi:10.1093/ehjopen/oeae076)

## Slide 1
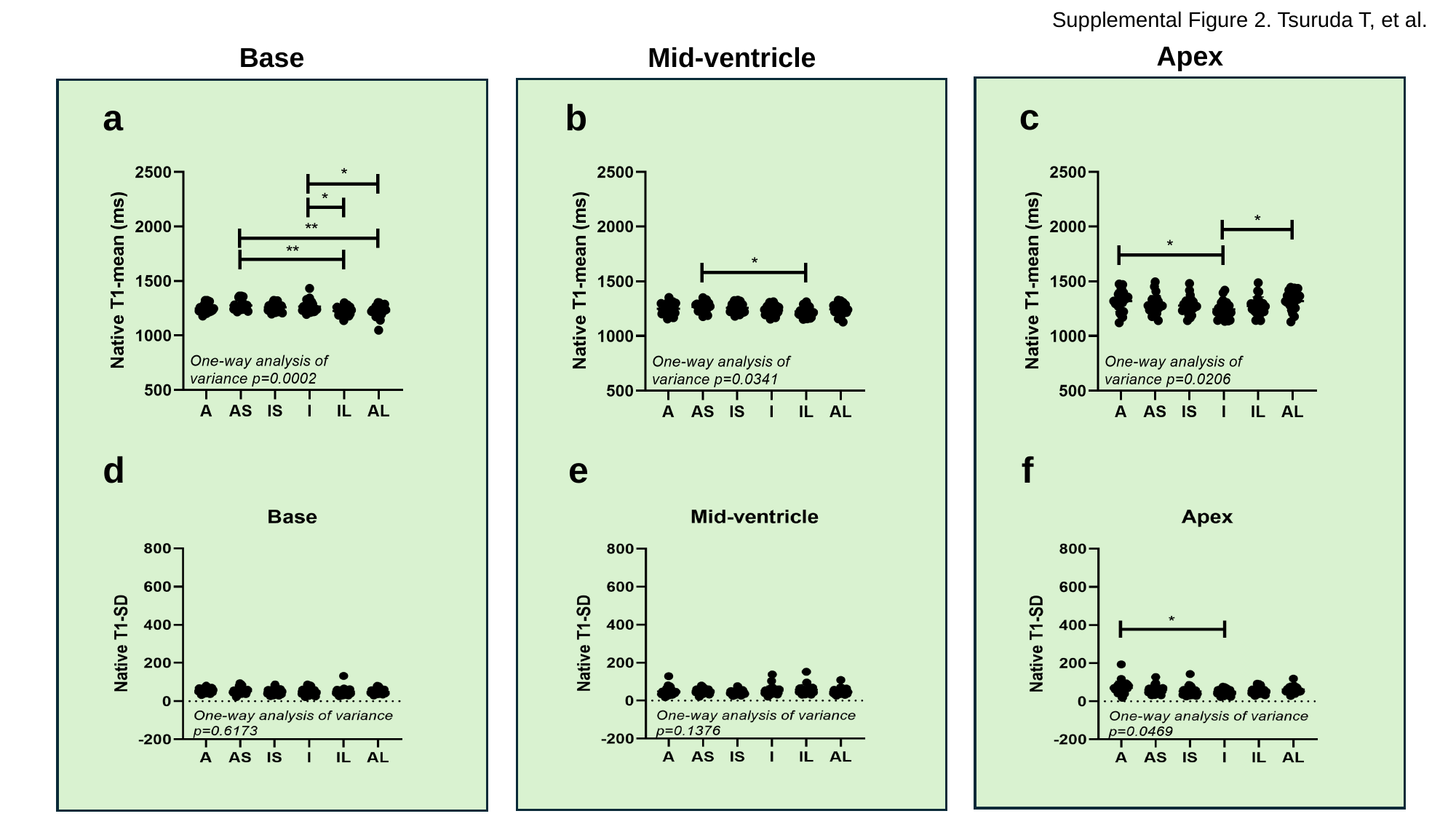

Supplemental Figure 2. Tsuruda T, et al.
Apex
Mid-ventricle
Base
c
a
b
d
f
e

Supplement: oeae076_Supplementary_Data [file oeae076_supplementary_data.zip › Supplemental Figure 2 Tsuruda T et al..pptx]

## Slide 1
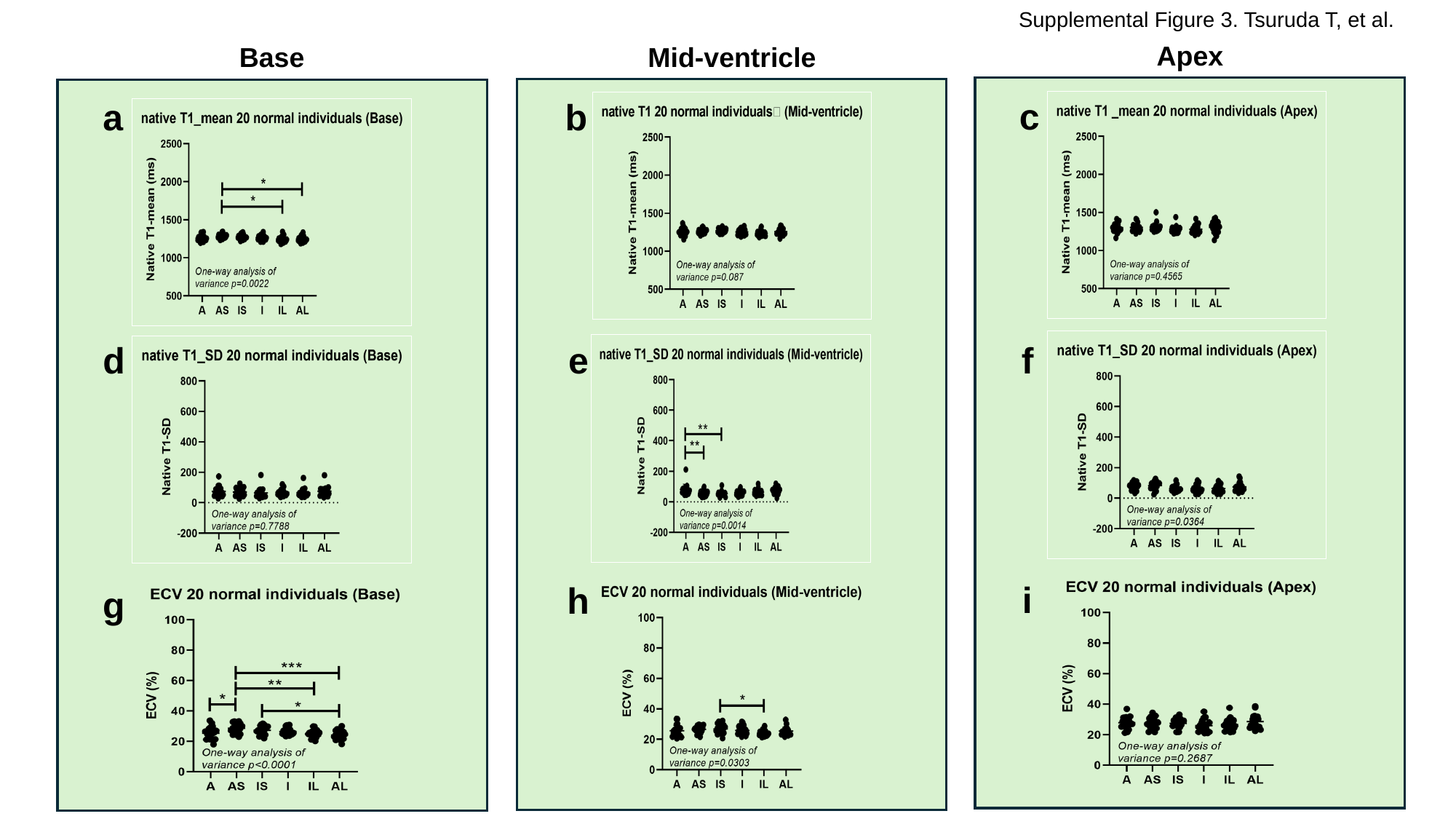

Supplemental Figure 3. Tsuruda T, et al.
Apex
Mid-ventricle
Base
c
a
b
d
f
e
i
h
g

Supplement: oeae076_Supplementary_Data [file oeae076_supplementary_data.zip › Supplemental Figure 3 Tsuruda T et al..pptx]
